# Supplementary material for: Prevalence of second mesiobuccal canal in maxillary molars of Iranian population: A systematic review with meta-analysis
Source: PLoS One. 2025 Jul 11;20(7):e0327006. doi: 10.1371/journal.pone.0327006 (PMC12250351; doi:10.1371/journal.pone.0327006)
Supplement: S3 Table — (DOCX) [file pone.0327006.s003.docx]

**S3 Table**. Joanna Briggs Institute (JBI) Critical Appraisal Checklist for studies reporting prevalence data.

| # | Question |
| --- | --- |
| Q1 | Was the sample frame appropriate to address the target population? |
| Q2 | Were study participants sampled in an appropriate way? |
| Q3 | Was the sample size adequate? |
| Q4 | Were the study subjects and the setting described in detail? |
| Q5 | Was the data analysis conducted with sufficient coverage of the identified sample? |
| Q6 | Were valid methods used for the identification of the condition? |
| Q7 | Was the condition measured in a standard, reliable way for all participants? |
| Q8 | Was there appropriate statistical analysis? |
| Q9 | Was the response rate adequate, and if not, was the low response rate managed appropriately? |
