# Supplementary material for: Prevalence of second mesiobuccal canal in maxillary molars of Iranian population: A systematic review with meta-analysis
Source: PLoS One. 2025 Jul 11;20(7):e0327006. doi: 10.1371/journal.pone.0327006 (PMC12250351; doi:10.1371/journal.pone.0327006)
Supplement: S5 Table — (DOCX) [file pone.0327006.s005.docx]

**S5 Table.** Excluded studies at the full text assessment phase with reason.

| **Reason for exclusion** | **Reference** |
| --- | --- |
| Iran's population was not assessed | 1. Murat Khan A, Yildirim C, Culha E, Demir E, Ciftci ME (2016) Detection of Second Mesiobuccal Canals in Maxillary First Molars Using a New Angle of Cone Beam Computed Tomography. Iranian Journal of Radiology 13(4), e31155. |
|  | 2. Smadi L, Khraisat A (2007) Detection of a second mesiobuccal canal in the mesiobuccal roots of maxillary first molar teeth. Oral Surg Oral Med Oral Pathol Oral Radiol Endod 103(3), e77-81. |
|  | 3. Hasan M, Raza Khan F (2014) Determination of Frequency of the Second Mesiobuccal Canal in the Permanent Maxillary First Molar Teeth with Magnification Loupes (× 3.5). International Journal of Biomedical Science 10(3): 201–207. |
| The second mesiobuccal canal was not evaluated | 4. Nazarimoghadam K, Farajian Zadeh N, Labbaf H, Kavosi A, Farajian Zadeh H (2019) Negotiation, Centering Ability and Transportation of Three Glide Path Files in Second Mesiobuccal Canals of Maxillary Molars: A CBCT Assessment. Iranian Endodontic Journal 14, 47-51.  5. Dalili Kajan Z, Taramsari M, Khosravi Fard N, Kanani M (2018) Accuracy of Cone-Beam Computed Tomography in Comparison with Standard Method in Evaluating Root Canal Morphology: An *In Vitro* Study. Iranian Endodontic Journal 13(2), 181–187. |
|  | 6. Razavian H, Sadeghian HR, Bagheri F (2014) Comparison of root canal displacement of maxillary maxillary first molar following preparation using stainless steel files by manual and rotational methods. Journal of Shahrekord University Medical Science 16(4), 21-27. |
|  | 7. Kuzekanani M, Haghani J, Izadi A, Keramati A (2014) Anatomic And Morphologic Evaluation Of Root Canal System Of Maxillary Third Molars In A Population In Kerman. Journal of Isfahan Dental School 10(3), 234-240. |
|  | 8. Shahravan A, Rekabi A, Shahabi H, Ashuri R, Mirzazadeh A, Rad M, Haghani J (2010) A Digital Stereomicroscopic Study of the Furcation Wall Thickness of Mesiobuccal Roots of Maxillary First and Second Molars. Iranian Endodontic Journal 5(2), 88-92. |
|  | 9. Sheikhi M, Zavar R, Maleki V (2012) Comparison of three periapical techniques in distinguishing the roots of maxillary second molars and preventing superimposition with zygomatic arch. Journal of Isfahan Dental School 8(3), 229-234. |
|  | 10. Esfahanian V, Ketabi M, Hafezibakhtiari M, Ashtari SH (2006) A Morphologic And Anatomic Evaluation Of Furcation Area In The Upper And Lower First Molars. Journal of Islamic Dental Association of Iran 2006 18(1), 13-20. |
|  | 11. Toodehzaeim MH, Mostafavi SMS (2016) Dental Arch Morphology in Iranian Population. Iranian Journal of Orthodontics 1(2):e5863. |
|  | 12. Haghanifar S, Moudi E, Madani Z, Farahbod F, Bijani A (2017) Evaluation of the Prevalence of Complete Isthmii in Permanent Teeth Using Cone-Beam Computed Tomography. Iranian Endodontic Journal 12(4), 426–431.  13. Mirzaie M, Tork Zaban P, Mohammadi V (2012) Cone-beam Computed Tomography Study of Root Canal in a Hamadani Population in Iran. Aviceanna Journal of Dental Research 4,93-99.  14. Mohammadzadeh Akhlaghi N, Ravandoust Y, Najafi M, Dadresanfar B (2012) An in vitro study of mesiobuccal root thickness of maxillary first molars. Iranian Endodontic Journal 7(1), 31-35.  15. Mirmohammadi H, Mahdi L, Partovi P, Khademi A, Shemesh H, Hassan B (2015) Accuracy of Cone-beam Computed Tomography in the Detection of a Second Mesiobuccal Root Canal in Endodontically Treated Teeth: An Ex Vivo Study. Journal of Endodontics 41(10), 1678-81.  16. Mohammadi Z, Asgari S, Shalavi S, Abott PV (2016) A Clinical Update on the Different Methods to Decrease the Occurrence of Missed Root Canals. Iranian Endodontic Journal 11(3):208-13.  17. Borna Z, Mohammadi Khoshrou M, Mirzaei H, Jabbari G (2020) Evaluation of Dentin Thickness around Second Mesiobuccal Canal in Maxillary First Molar Using Cone Beam Computed Tomography (CBCT). International Journal of Scientific Research 2, 1-5. |
| The type of study was a review | 18. Ghasemi N, Rahimi S, Shahi SH, Samiei M, Reyhani MF, Ranjkesh B (2016) A Review on Root Anatomy and Canal Configuration of the Maxillary Second Molars. Iranian Endodontic Journal 12(1), 1–9. |
|  | 19. Naseri M, Kharazifard MJ, Hosseinpour S (2016) Canal configuration of mesiobuccal roots in permanent maxillary first molars in Iranian population: A systematic review. Journal of Dentistry of Tehran University of Medical Sciences 13(6): 438–447. |
