# Supplementary material for: Prevalence of second mesiobuccal canal in maxillary molars of Iranian population: A systematic review with meta-analysis
Source: PLoS One. 2025 Jul 11;20(7):e0327006. doi: 10.1371/journal.pone.0327006 (PMC12250351; doi:10.1371/journal.pone.0327006)
Supplement: S6 Table — (DOCX) [file pone.0327006.s006.docx]

**S6 Table**. Quality assessment of included studies of maxillary first molars according to Joanna Briggs Institute (JBI) Critical Appraisal Checklist for studies reporting prevalence data.

| **Author, year** | **Q1** | **Q2** | **Q3** | **Q4** | **Q6** | **Q7** | **Q8** | **Overall score** |
| --- | --- | --- | --- | --- | --- | --- | --- | --- |
| Sharifi, 2023 | Y | Y | Y | Y | Y | Y | Y | 100 |
| Namdar, 2023 | Y | Y | Y | Y | Y | Y | Y | 100 |
| Khademi (A), 2022 | Y | Y | Y | Y | Y | Y | Y | 100 |
| Dibaji, 2022 | Y | Y | Y | Y | Y | Y | Y | 100 |
| Karkehabadi, 2022 | N | Y | Y | Y | Y | Y | Y | 86 |
| Esmaeilian, 2021 | Y | Y | Y | Y | Y | Y | Y | 100 |
| Nikkerdar 2020 | N | Y | Y | Y | Y | Y | Y | 86 |
| Tafakhori, 2018 | Y | Y | N | Y | Y | Y | Y | 86 |
| Khosravifard, 2018 | Y | Y | Y | Y | Y | Y | Y | 100 |
| Ghoncheh, 2017 | N | Y | Y | Y | Y | Y | Y | 86 |
| Zand (A), 2017 | Y | Y | Y | Y | Y | Y | Y | 100 |
| Ghaznavi, 2017 | Y | Y | Y | Y | Y | Y | Y | 100 |
| Khademi, 2016 | Y | Y | Y | Y | Y | Y | Y | 100 |
| Naseri, 2016 | Y | Y | Y | Y | Y | Y | Y | 100 |
| Faramarzi, 2015 | Y | Y | Y | Y | Y | Y | Y | 100 |
| Ezoddini Ardakani, 2014 | Y | Y | N | Y | Y | Y | Y | 86 |
| Rouhani, 2014 | N | Y | Y | Y | Y | Y | Y | 86 |
| Rezaeian, 2018 | N | Y | Y | Y | Y | Y | Y | 86 |
| Naseri, 2015 | Y | Y | N | Y | Y | Y | Y | 86 |
| Adel, 2009 | N | Y | Y | N | Y | Y | Y | 72 |
| Shahi, 2007 | Y | Y | Y | Y | Y | Y | Y | 100 |
| Hasheminia (A), 2005 | Y | Y | Y | Y | Y | Y | Y | 100 |
| Sadeghi, 2004 | Y | Y | Y | Y | Y | Y | Y | 100 |
| Zand (B), 2017 | Y | Y | Y | Y | Y | Y | Y | 100 |
| Safi, 2000 | N | Y | N | Y | Y | Y | Y | 72 |
| Ashofteh Yazdi, 2005 | N | Y | Y | Y | Y | Y | Y | 86 |
| Hasheminia (B), 2005 | Y | Y | Y | Y | Y | Y | Y | 100 |
| Ghorbanzadeh (A), 2009 | Y | Y | N | Y | Y | Y | Y | 86 |
| Parirokh, 2023 | Y | Y | Y | Y | Y | Y | Y | 100 |
| Khademi (C), 2022 | Y | Y | Y | Y | Y | Y | Y | 100 |
| Ghorbanzadeh (B), 2009 | Y | Y | N | Y | Y | Y | Y | 86 |
| Ghorbanzadeh (C), 2009 | Y | Y | N | Y | Y | Y | Y | 86 |
| Khademi (B), 2022\ | Y | Y | Y | Y | Y | Y | Y | 100 |

Two criteria on coverage bias (Q5) and response rate (Q9) were not considered. N:No; Y:Yes
