# Supplementary material for: Prevalence of second mesiobuccal canal in maxillary molars of Iranian population: A systematic review with meta-analysis
Source: PLoS One. 2025 Jul 11;20(7):e0327006. doi: 10.1371/journal.pone.0327006 (PMC12250351; doi:10.1371/journal.pone.0327006)
Supplement: S7 Table — (DOCX) [file pone.0327006.s007.docx]

**S7 Table**. Quality assessment of included studies of maxillary second molars according to Joanna Briggs Institute (JBI) Critical Appraisal Checklist for studies reporting prevalence data.

| **Author, year** | **Q1** | **Q2** | **Q3** | **Q4** | **Q6** | **Q7** | **Q8** | **Overall score** |
| --- | --- | --- | --- | --- | --- | --- | --- | --- |
| Namdar, 2023 | Y | Y | Y | Y | Y | Y | Y | 100 |
| Karkehabadi, 2022 | N | Y | Y | Y | Y | Y | Y | 86 |
| Esmaeilian, 2021 | Y | Y | Y | Y | Y | Y | Y | 100 |
| Nikkerdar, 2020 | N | Y | Y | Y | Y | Y | Y | 86 |
| Naseri, 2018 | Y | Y | Y | Y | Y | Y | Y | 100 |
| Khosravifard., 2018 | Y | Y | Y | Y | Y | Y | Y | 100 |
| Ghoncheh, 2017 | N | Y | Y | Y | Y | Y | Y | 86 |
| Zand (A), 2017 | Y | Y | Y | Y | Y | Y | Y | 100 |
| Khademi, 2016 | Y | Y | Y | Y | Y | Y | Y | 100 |
| Rouhani, 2014 | N | Y | Y | Y | Y | Y | Y | 86 |
| Naseri, 2015 | Y | Y | N | Y | Y | Y | Y | 86 |
| Zarei, 2009 | Y | Y | Y | Y | Y | Y | Y | 100 |
| Hasheminia (A), 2005 | Y | Y | Y | Y | Y | Y | Y | 100 |
| Sadeghi, 2004 | Y | Y | Y | Y | Y | Y | Y | 100 |
| Zand (B), 2017 | Y | Y | Y | Y | Y | Y | Y | 100 |
| Safi, 2000 | N | Y | N | Y | Y | Y | Y | 72 |
| Ghorbanzadeh (A), 2009 | Y | Y | N | Y | Y | Y | Y | 86 |
| Parirokh, 2023 | Y | Y | Y | Y | Y | Y | Y | 100 |
| Ghorbanzadeh (B), 2009 | Y | Y | N | Y | Y | Y | Y | 86 |
| Ghorbanzadeh (C), 2009 | Y | Y | N | Y | Y | Y | Y | 86 |

Two criteria on coverage bias (Q5) and response rate (Q9) were not considered. N:No; Y:Yes
