# Supplementary material for: Prevalence of second mesiobuccal canal in maxillary molars of Iranian population: A systematic review with meta-analysis
Source: PLoS One. 2025 Jul 11;20(7):e0327006. doi: 10.1371/journal.pone.0327006 (PMC12250351; doi:10.1371/journal.pone.0327006)
Supplement: S8 Table — (DOCX) [file pone.0327006.s008.docx]

**S8 Table.** Overall MB2 prevalence in maxillary first and second molars in the different counties.

| **Second molar** | **First molar** | **Country** |
| --- | --- | --- |
| - | 93.5% | Turkiye (1) |
|  | 67.3% | Thailand (2) |
| 34.32% | - | Brazil (3) |
|  | 24.9% | Uganda (4) |
| 34.39% | - | Korea (5) |
| - | 45% | Saudi Arabia (6) |
| 50% | - | India (7) |
| - | 70.1% | Spain (8) |
| - | 79.6% | Italy (8) |
| - | 84% | Mexico (8) |
| - | 79.8% | Kuwait (8) |
| - | 57.8% | Costa Rica (8) |
| - | 61.9% | India (9) |
| 29.9% | - | China (10) |
| 29.8% | 51.3% | Brunei (11) |

**Reference**

1. Sert S, Bayirli GS. Evaluation of the root canal configuration of the mandibular and maxillary permanent teeth by gender in the Turkish population. J Endod. 2004 ;30(6):391-8.

2.Alavi AM, Opasanon A, Ng YI, Gulabivala K. Root and canal morphology of Thai maxillary molars. Int Endod J. 2002;35(5):478-85.

3. Silva EJ, Nejaim Y, Silva AI, Haiter-Neto F, Zaia AA, Cohenca N. Evaluation of the root canal configuration of maxillary molars in a Brazilian population using cone-beam computed tpmpgraphic imagine: an in vivo. J Endod. 2014 ;40(2):173-6.

4. Rwenyonyi CM, Kutesa AM, Muwazi LM, Buwembo W. Root and canal morphology of maxillary first and second permanent molar teeth in a Ugandan population. . Int Endod J. 2007;40(9):679-83.

5. Kim Y, Lee SJ, Woo J. morphology of maxillary first and second molars analyzed by cone-beam computed tpmpgraphy in a Korean population: variations in the number of roots and canals and the incidence of fusion. J Endod. 2012 ;38(8):1063-8.

6. Agwan A S, Sheikh Z, Rashid H. Canal Configuration And The Prevalence Of Second Mesiobuccal Canal In Maxillary First Molar Of A Saudi Sub-Population. Journal the Pakistan Dental association. 2015;24(4):1680-2292.

7. Neelakantan P, Subbarao C, Ahuja R, Subbarao CV, Gutmann JL. Cone-beam computed tpmpgraphy study of root and canal morphology of maxillary first and second molars in an Indian population. J Endod. 2010 ;36(10):1622-7.

8. Martins JNR, Alkhawas MAM, Altaki Z, et al. Worldwide Analyses of Maxillary First Molar Second Mesiobuccal Prevalence: A Multicenter Cone-beam Computed Tomographic Study. J Endod. 2018;44(11):1641-1649.e1.

9. Kewalramani R, Murthy CS, Gupta R. The second mesiobuccal canal in three-rooted maxillary first molar of Karnataka Indian sub-populations: A cone-beam computed tomography study. J Oral Biol Craniofac Res. 2019;9(4):347-35110.

10. Wu D, Zhang G, Liang R, Zhou G, Wu Y, Sun C, Fan W. Root and canal morphology of maxillary second molars by cone-beam computed tomography in a native Chinese population. J Int Med Res. 2017;45(2):830-842.

11. Onn HY, Sikun MSYA, Abdul Rahman H, Dhaliwal JS. Prevalence of mesiobuccal-2 canals in maxillary first and second molars among the Bruneian population-CBCT analysis. BDJ Open. 2022 19;8(1):32.
