# Supplementary material for: Prevalence of second mesiobuccal canal in maxillary molars of Iranian population: A systematic review with meta-analysis
Source: PLoS One. 2025 Jul 11;20(7):e0327006. doi: 10.1371/journal.pone.0327006 (PMC12250351; doi:10.1371/journal.pone.0327006)
Supplement: S9 Table — (DOCX) [file pone.0327006.s009.docx]

**S9 Table**. Overall MB2 root canal prevalence in maxillary first molars according to voxel size

| **Year** | **Prevalence (%)** | **Voxel size (µm)** | **City** | **Author** |
| --- | --- | --- | --- | --- |
| 2023  2023  2022  2022  2022  2021  2020 | 88  71.3  70.4  50.1  70  67.5  51 | 150  75  100  300  200  NR  150 | Kermanshah  Mazandaran  Isfahan  Tehran  Hamedan  Isfahan  Kermanshah | Sharifi et al. (1)  Namdar et al. (2)  Khademi (A) et al. (3)  Dibaji et al. (4)  Karkehabadi et al. (5)  Esmaeilian et al. (6)  Nikkerdar et al. (7) |
| 2018 | 63 | 150 | Rafsanjan | Tafakhori et al. (8) |
| 2018 | 45 | 200-240 | Rasht | Khosravifard et al. (9) |
| 2017 | 46 | 300 | Tehran | Ghonch et al. (10) |
| 2017 | 55 | 127 | Tabriz | Zand et al. (11) |
| 2017 | 41 | 200 | Urmia | Ghaznavi et al. (12) |
| 2016 | 70 | 150 | Isfahan | Khademi et al. (13) |
| 2016 | 87 | 200 | Tehran | Naseri et al. (14) |
| 2015 | 69 | NR | Hamedan | Faramarzi et al. (15) |
| 2014 | 60 | 320 | Yazd | Ezoddini Ardakani et al. (16) |
| 2014 | 54 | 160 | Mashhad | Rouhani et al. (17) |

NR: not reported

**Reference**

1.Sharifi R, Nikkerdar N, Mohammadzaki F, Fallahnia N. CBCT evaluation of undetected second mesiobuccal canals and configuration on the prevalence of periapical lesions in root canal-treated maxillary first molars. International Journal of Medical Dentistry. 2023;27(2):257-262.

2.Namdar P, Molania T, Hoshyari N, Lotfizadeh A, Alimohammadi M, Khojastehfar M, Haddadi Kohsar A. Evaluation of root and canal morphology of maxillary first and second molars by cone beam computed tomography in a northern Iranian population. Journal of Research in Dental and Maxillofacial Sciences. 2023;8(4):265-273.

3.Khademi A, Saatchi M, Sheikhi M, Soltani MM, Moradi S. In Vitro diagnostic accuracy and agreement of dental microscope and cone-beam computed tomography in comparison with microcomputed tomography for detection of the second mesiobuccal canal of maxillary first molars. Hidawi. 2022,Article ID 1493153, 6 pages.

4.Dibaji F, Shariati R, Moghaddamzade B, Mohammadian F, Sooratgar A, Kharazifard M. Evaluation of the relationship between buccolingual width of mesiobuccal root and root canal morphology of maxillary first molars by cone-beam computed tomography. Dental Research Journal. 2022;19:5.

5.Karkehabadi H, Shokri A, Cheraghi Z, Mombeini A,Ghasemi L, Ahmadyani E. Root canal morphology of maxillary first and second molars and their relation with anatomical landmarks using cone-beam computed tomography. International Journal of Clinical Dentistry. 2022;15(4)729-738.

6.Esmaeilian A, Torkzadeh A, Mortaheb A, Zakariaee Juybari A. The examination of root morphology of the maxillary first and second molars using cone beam computed tomography. Journal of Isfahan Dental School. 2021;17(3):329-336.

7.Nikkerdar N, Asnaashari M, Karimi A, Araghi S, Seifitabar SH, Golshah A. Root and canal morphology of maxillary teeth in an Iranian subpopulation residing in western iran using cone-beam computed tomography. Iranian Endodontic Journal. 2020;15(1):31-37.

8.Tafakhori Z, Sheikh Fathollahi M. A survey on anatomy of mesiobuccal root canal in permanent maxillary first molar using CBCT in rafsanjan in 2018: A descriptive study. J Rafsanjan Univ Med Sci. 2020;19(4):357-68.

9.Khosravifard N, DliliKajan Z, Hasanpoor H. Cone Beam Computed Tomographic Survey of the Mesiobuccal Root Canal Anatomy in the Maxillary First and Second Molar. European Journal of Dentistry. 2018;12(3):422-427.

10.Ghoncheh Z, MoghaddamZade B, Kharazifard M J. Root Morphology of the Maxillary First and Second Molars in an Iranian Population Using Cone Beam Computed Tomography. J Dent(Tehran). 2017;14(3):115-122.

11.Zand V, Mokhtari H, Zonouzi HR, Shojaei SN. Root canal morphologies of mesiobuccal roots of maxillary molars using cone beam computed tomography and priapical radiographic technique in an Iranian population. The Journal of Contemporary Dental Practice. 2017;18(9):745-749.

12.Ghaznavi A, Ilbeigi Diarjan A, Abaszadeh A, Jafary Heidarloo M, Esmaili Moghaddam M. Prevalence of second mesiobuccal canal in maxillary canal in maxillary first molar by cone beam computed tomography in patients referred to radiology centers of urumia in 2011-2014. The Journal of Urmia University of Medical Sciences. 2017;28(9):528 ISSN:1027-3727.

13.Khademi A, Zamani Nasr A, Bahreinian Z, Mehdizadeh M, Khazaei S. Root Morphology and Canal Configuration of First and Second Maxillary Molars in a Selected Iranian Population: A Cone-Beam Computed Tomography Evaluation. Iranian Endodontic Journal. 2017;12(3):288-292.

14. Naseri M, Safi Y, Akbarzadeh Baghban A, Khayat A, Eftekhar L. Survey of anatomy and root canal morphology of maxillary first molar regarding age and gender in an Iranian population using cone-beam computed tomography. Iranian Endodontic Journal. 2016;11(4):298-303.

15.Faramarzi F, Vossoghi M, Shokri A, Shams B, Vossoghi M, Khoshbin E. Cone beam computed tomography study of root and canal morphology of maxillary first molar in an Iranian population. Avicenna J Dent Res. 2015;7(1):e24038.

16.Ezoddini Ardakani F, Mirhosseini F, Karrabi M, Mokhtari N et al. Analysis anatomy of mesio-buccal root canal in maxillary first permanent molars using CBCT technique (In vitro). Journal of Shahid Sadoughi University of Medical Sciences. 2014;22(2):1010-9.

17.Rouhani A, Bagherpour A, Akbari M et al. Cone-Beam Computed Tomography Evaluation of Maxillary First and Second Molars in Iranian Population. A Morphological. Iranian Endodontic Journal. 2014;9(3):190-194.
