# Supplementary material for: Prevalence of second mesiobuccal canal in maxillary molars of Iranian population: A systematic review with meta-analysis
Source: PLoS One. 2025 Jul 11;20(7):e0327006. doi: 10.1371/journal.pone.0327006 (PMC12250351; doi:10.1371/journal.pone.0327006)
Supplement: S10 Table — (DOCX) [file pone.0327006.s010.docx]

**S10 Table.** Overall MB2 root canal prevalence in maxillary second molars according to voxel size

| **Year** | **Prevalence (%)** | **Voxel size (µm)** | **City** | **Author** |
| --- | --- | --- | --- | --- |
| 2023  2022  2021  2020 | 37.1  35.5  23.5  51 | 75  200  NR  150 | Mazandaran  Hamedan  Isfahan  Kermanshah | Namdar et al. (1)  Karkehabadi et al. (2)  Esmaeilian et al. (3)  Nikkerdar et al. (4) |
| 2018  2018 | 68  45 | 200  200-240 | Tehran  Rasht | Naseri et al. (5)  Khosravifard et al. (6) |
| 2017 | 46 | 300 | Tehran | Ghonch et al. (7) |
| 2017 | 55 | 127 | Tabriz | Zand et al. (8) |
| 2016 | 70 | 150 | Isfahan | Khademi et al. (9) |
| 2014 | 54 | 160 | Mashhad | Rouhani et al. (10) |

NR: not reported

**Reference**

1.Namdar P, Molania T, Hoshyari N, Lotfizadeh A, Alimohammadi M, Khojastehfar M, Haddadi Kohsar A. Evaluation of root and canal morphology of maxillary first and second molars by cone beam computed tomography in a northern Iranian population. Journal of Research in Dental and Maxillofacial Sciences. 2023;8(4):265-273.

2.Karkehabadi H, Shokri A, Cheraghi Z, Mombeini A,Ghasemi L, Ahmadyani E. Root canal morphology of maxillary first and second molars and their relation with anatomical landmarks using cone-beam computed tomography. International Journal of Clinical Dentistry. 2022;15(4)729-738.

3.Esmaeilian A, Torkzadeh A, Mortaheb A, Zakariaee Juybari A. The examination of root morphology of the maxillary first and second molars using cone beam computed tomography. Journal of Isfahan Dental School. 2021;17(3):329-336.

4.Nikkerdar N, Asnaashari M, Karimi A, Araghi S, Seifitabar SH, Golshah A. Root and canal morphology of maxillary teeth in an Iranian subpopulation residing in western iran using cone-beam computed tomography. Iranian Endodontic Journal. 2020;15(1):31-37.

5.Naseri M, Mozayeni MA, Safi Y, Heidarnia M, Akbarzadeh Baghban A, Norouzi N. Root canal morphology of maxillary second molars according to age and gender in a selected Iranian population: A cone-beam computed tomography evaluation. Iranian Endodontic Journal. 2018;13(3):373-380.

6.Khosravifard N, DliliKajan Z, Hasanpoor H. Cone Beam Computed Tomographic Survey of the Mesiobuccal Root Canal Anatomy in the Maxillary First and Second Molar. European Journal of Dentistry. 2018;12(3):422-427.

7.Ghoncheh Z, MoghaddamZade B, Kharazifard M J. Root Morphology of the Maxillary First and Second Molars in an Iranian Population Using Cone Beam Computed Tomography. J Dent(Tehran). 2017;14(3):115-122.

8.Zand V, Mokhtari H, Zonouzi HR, Shojaei SN. Root canal morphologies of mesiobuccal roots of maxillary molars using cone beam computed tomography and priapical radiographic technique in an Iranian population. The Journal of Contemporary Dental Practice. 2017;18(9):745-749.

9.Khademi A, Zamani Nasr A, Bahreinian Z, Mehdizadeh M, Khazaei S. Root Morphology and Canal Configuration of First and Second Maxillary Molars in a Selected Iranian Population: A Cone-Beam Computed Tomography Evaluation. Iranian Endodontic Journal. 2017;12(3):288-292.

10.Rouhani A, Bagherpour A, Akbari M et al. Cone-Beam Computed Tomography Evaluation of Maxillary First and Second Molars in Iranian Population. A Morphological. Iranian Endodontic Journal. 2014;9(3):190-194.
