# Supplementary material for: Prevalence of second mesiobuccal canal in maxillary molars of Iranian population: A systematic review with meta-analysis
Source: PLoS One. 2025 Jul 11;20(7):e0327006. doi: 10.1371/journal.pone.0327006 (PMC12250351; doi:10.1371/journal.pone.0327006)
Supplement: Attachment — Submitted filename: Response to Reviewers.docx [file pone.0327006.s012.docx]

| **Editor comments** | | |
| --- | --- | --- |
| **Comments** | **Reply** | **Reference** |
| PONE-D-24-24127  Prevalence of Second Mesiobuccal Canal in Maxillary Molars of Iranian Population: Systematic Review with Meta-Analysis  PLOS ONE  Dear Dr. Behdarvandi,  Thank you for submitting your manuscript to PLOS ONE. After careful consideration, we feel that it has merit but does not fully meet PLOS ONE’s publication criteria as it currently stands. Therefore, we invite you to submit a revised version of the manuscript that addresses the points raised during the review process. | Thanks for the valuable comments. | - |
| Figures 3 to 5 need more explanation in the text. | Additional explanation was included in the results and discussion section. Please note that Figure 4 has been removed in accordance with comment #6 from the second reviewer. | Page 9, 148-51.  Page 10, lines 171-5.  Page 11, lines 181-4.  Page 15, line 272-5. |
| Please submit your revised manuscript by Dec 14 2024 11:59PM. If you will need more time than this to complete your revisions, please reply to this message or contact the journal office at [plosone@plos.org](mailto:plosone@plos.org). When you're ready to submit your revision, log on to <https://www.editorialmanager.com/pone/> and select the 'Submissions Needing Revision' folder to locate your manuscript file.  Please include the following items when submitting your revised manuscript:   - A rebuttal letter that responds to each point raised by the academic editor and reviewer(s). You should upload this letter as a separate file labeled 'Response to Reviewers'. - A marked-up copy of your manuscript that highlights changes made to the original version. You should upload this as a separate file labeled 'Revised Manuscript with Track Changes'. - An unmarked version of your revised paper without tracked changes. You should upload this as a separate file labeled 'Manuscript'.   If you would like to make changes to your financial disclosure, please include your updated statement in your cover letter. Guidelines for resubmitting your figure files are available below the reviewer comments at the end of this letter.  If applicable, we recommend that you deposit your laboratory protocols in [protocols.io](http://protocols.io/) to enhance the reproducibility of your results. Protocols.io assigns your protocol its own identifier (DOI) so that it can be cited independently in the future. For instructions see: [https://journals.plos.org/plosone/s/submission-guidelines#loc-laboratory-protocols](https://track.editorialmanager.com/CL0/https:%2F%2Fjournals.plos.org%2Fplosone%2Fs%2Fsubmission-guidelines%23loc-laboratory-protocols/1/010f0192dfdfafbc-0183165e-395b-49e1-acf3-2b8ecf144d31-000000/NI3k6MU9mjh_mTqgjuWO0gEpDalrk1np5w2tpESLJW4=183). Additionally, PLOS ONE offers an option for publishing peer-reviewed Lab Protocol articles, which describe protocols hosted on [protocols.io](http://protocols.io/). Read more information on sharing protocols at [https://plos.org/protocols?utm_medium=editorial-email&utm_source=authorletters&utm_campaign=protocols](https://track.editorialmanager.com/CL0/https:%2F%2Fplos.org%2Fprotocols%3Futm_medium=editorial-email%26utm_source=authorletters%26utm_campaign=protocols/1/010f0192dfdfafbc-0183165e-395b-49e1-acf3-2b8ecf144d31-000000/aQ3KvNR54qhEat2mMB-AgzwWgWoqCO3KVrWYjPXZmb8=183).  We look forward to receiving your revised manuscript.  Kind regards,  Mohammed Nasser Alhajj, BDS, MClinDent, PhD Academic Editor PLOS ONE | Thanks for your comments. The file namely “'Response to Reviewers'”, “Revised Manuscript with Track Changes” and “Manuscript” were uploaded.  The financial disclosure was not changed.  Since the study was systematic review and meta-analysis, laboratory protocols was not applicable. | - |
| **Journal Requirements:** | | |
| When submitting your revision, we need you to address these additional requirements. | We appreciated and considered the comments. | - |
| 1. Please ensure that your manuscript meets PLOS ONE's style requirements, including those for file naming. The PLOS ONE style templates can be found at https://journals.plos.org/plosone/s/file?id=wjVg/PLOSOne_formatting_sample_main_body.pdf and ... | They were included, thank you. | - |
| 2. Please note that funding information should not appear in any section or other areas of your manuscript. We will only publish funding information present in the Funding Statement section of the online submission form. Please remove any funding-related text from the manuscript. | The sentence was removed. | - |
| 3. Thank you for stating the following financial disclosure: “SM.H received the grant by Isfahan University of Medical Sciences (#3400125).”  Please state what role the funders took in the study. If the funders had no role, please state: "The funders had no role in study design, data collection and analysis, decision to publish, or preparation of the manuscript." If this statement is not correct you must amend it as needed. Please include this amended Role of Funder statement in your cover letter; we will change the online submission form on your behalf. | Done, thank you. | - |
| 4. We note that your Data Availability Statement is currently as follows: “All relevant data are within the manuscript and in Supporting Information files.”  Please confirm at this time whether or not your submission contains all raw data required to replicate the results of your study. Authors must share the “minimal data set” for their submission. PLOS defines the minimal data set to consist of the data required to replicate all study findings reported in the article, as well as related metadata and methods (<https://journals.plos.org/plosone/s/data-availability#loc-minimal-data-set-definition>). For example, authors should submit the following data: - The values behind the means, standard deviations and other measures reported; - The values used to build graphs; - The points extracted from images for analysis. Authors do not need to submit their entire data set if only a portion of the data was used in the reported study. If your submission does not contain these data, please either upload them as Supporting Information files or deposit them to a stable, public repository and provide us with the relevant URLs, DOIs, or accession numbers. For a list of recommended repositories, please see <https://journals.plos.org/plosone/s/recommended-repositories>. If there are ethical or legal restrictions on sharing a de-identified data set, please explain them in detail (e.g., data contain potentially sensitive information, data are owned by a third-party organization, etc.) and who has imposed them (e.g., an ethics committee). Please also provide contact information for a data access committee, ethics committee, or other institutional body to which data requests may be sent. If data are owned by a third party, please indicate how others may request data access. | Thanks for your comment.  All data is available via <https://doi.org/10.6084/m9.figshare.28070612>. | - |
| 5. PLOS requires an ORCID iD for the corresponding author in Editorial Manager on papers submitted after December 6th, 2016. Please ensure that you have an ORCID iD and that it is validated in Editorial Manager. To do this, go to ‘Update my Information’ (in the upper left-hand corner of the main menu), and click on the Fetch/Validate link next to the ORCID field. This will take you to the ORCID site and allow you to create a new iD or authenticate a pre-existing iD in Editorial Manager. | Done, thank you. | - |
| 6. Please ensure that you refer to Figure 3 in your text as, if accepted, production will need this reference to link the reader to the figure. | Done, thank you. | Page 9, line 148.  Page 10, line 175. |
| 7. As required by our policy on Data Availability, please ensure your manuscript or supplementary information includes the following: A numbered table of all studies identified in the literature search, including those that were excluded from the analyses. For every excluded study, the table should list the reason(s) for exclusion. If any of the included studies are unpublished, include a link (URL) to the primary source or detailed information about how the content can be accessed. A table of all data extracted from the primary research sources for the systematic review and/or meta-analysis. The table must include the following information for each study: Name of data extractors and date of data extraction Confirmation that the study was eligible to be included in the review. All data extracted from each study for the reported systematic review and/or meta-analysis that would be needed to replicate your analyses. If data or supporting information were obtained from another source (e.g. correspondence with the author of the original research article), please provide the source of data and dates on which the data/information were obtained by your research group. If applicable for your analysis, a table showing the completed risk of bias and quality/certainty assessments for each study or outcome. Please ensure this is provided for each domain or parameter assessed. For example, if you used the Cochrane risk-of-bias tool for randomized trials, provide answers to each of the signalling questions for each study. If you used GRADE to assess certainty of evidence, provide judgements about each of the quality of evidence factor. This should be provided for each outcome. An explanation of how missing data were handled. This information can be included in the main text, supplementary information, or relevant data repository. Please note that providing these underlying data is a requirement for publication in this journal, and if these data are not provided your manuscript might be rejected. | All data is available via the tables, available supporting information and Figshare (<https://doi.org/10.6084/m9.figshare.28070612>.) | Tables 1 and 2.  Tables S2, S3, S4 and Sd. |
| Dear authors  Thank you for submitting your manuscript to PLOS One journal. The manuscript has been now reviewed by 2 independent reviewers who had some comments that should be addressed. Kindly go through the comments and respond to all comments point by point.  We are looking forward to receiving the revised version.  Regards, | Thanks for valuate comments. The manuscript was revised. | - |
| **Reviewer #1** | | |
| Abstract ok | Thank you. | - |
| Key words  better to be in alphabetical order | Done, thank you. | Page 5, lines 45-6. |
| Introduction: In general, not tight enough and need improvements, specially citing the recent and related references. Better to go shortly highlighting what has been done in your population and how many studies and technique used in them. then, the limitation present the current studies in your population, and clear the aim of the study. | The references were updated with more recent and relevant sources, thank you. | Page 2, lines 54-55. |
| Missing the second mesiobuccal (MB2) canal was suggested as one of the most common failure reasons for root canal treatment in maxillary molars (1).  The reference is not related at all, you should bring some reference from outcome studies or missed canals, or failure, Extra….. | Done, thank you. | Page 2, lines 67-70. |
| Although most studies have shown the presence of the MB2 canal, there is no general agreement about its prevalence in different populations (5).  Not related reference. Get one from gender differences, race differences and so on. Better to get some systematic review studies reporting these variables. | Done, thank you. | Page 2, lines 57-63. |
| The prevalence of MB2 in maxillary molars ranges from 10% to 95% (6),  This is a big statement, its better to mentions if is it worldwide or what exactly, also bring a new related reference, check Martins…. | The sentence was deleted, thanks for your insightful comment. | - |
| which might be related to the method of assessment and the race, age, and gender of the study population (5).    Same note here the reference…. | Done, thank you. | Page 2, lines 61-3. |
| Materials and methods ok | Thank you. |  |
| RESULTS:  In contrast, the lowest prevalence of MB2 canal was related to the direct vision method with a prevalence of 27% (95%CI, 13_40; I2=0%), followed by the PA radiography method with a prevalence of 15% (95%CI, -10_40; I2=91.17%)  SHOULD BE THE OTHER WAY AROUND | Done, thank you. | Page 9, lines 145-7. |
| In contrast, the lowest prevalence was related to the direct vision method with a prevalence of 16% (95%CI, 4_27;I2=0%), followed by the PA radiography method with a prevalence of 4% (95% CI, -8_17;I2=40.52%)  SHOULD BE THE OTHER WAY AROUND | Done, thank you. | Page 10, lines 168-70 |
| DISCUSSION:  Generally not bad but need to be improved, Compare your pool outcome with other international studies and other sys reviews as well | Done, thank you. | Page 12, lines 198-203 |
| You did good in explaining the variations between the pool of studies you collected | Thank you. | - |
| No limitations was mentioned in your study? | A paragraph addressing the limitations has been added. Thank you for your comment. | Page 14, lines 265-75. |
| In the present study, the pooled prevalence of the MB2 canal in the maxillary first molar (60%) was higher than the second molar (33%), which was similar to other studies (35). Poor reference, better to compare with other sys reviews worldwide, or mention more than one country to compare with….. | Thanks for your valuable comment, done. | Page 12, lines 198-203. |
| In various populations, a range of 25% to 93.5% and 34% to 50% have been reported for the first and second molars, respectively (S5 Table). This different range in the countries can be due to various evaluation methods, racial groups, or geographical regions. Where is the reference? Most references in table S5 is old and well selected | The S5 table (S7 table in the revision) has been updated with recent studies, and a reference was added. Thank you for your insightful comment. | Page 12, lines 198-203. |
| Natural selection is also associated with phenotype changes (36). Large tooth size may be maintained as a dominant natural selection (African population), while the reduction in tooth size occurs in the absence of such force (37). What do you mean by these sentences, better to clarify in the texts… | The entire of paragraph was revised, thank you. | Page 12, lines 204-12. |
| Further implication: Future studies are suggested by accurately reporting the age, gender, and geographic location of the teeth, as well as using modern techniques, such as the microscope, loupe, and micro-CT. CBCT IN VIVO IS THE BEST TO DESCRIBE ALL THE VARIABLES INCLUDING RIGHT AND LET, AGE, GENDER AND EXTEA… Micro-CT is very precise but it can be traumatic and one of its imitation is the number of sample Versus CBCT Where you can get a very big sample | The sentences was revised, thank you. | Page 12, lines 280-2. |
| Conclusion: The prevalence of the MB2 canal was higher in the first molar (60%) than in the second molar (33%) of the maxilla, and the prevalence of the MB2 canal depended on the assessment method.  According to your study and limitations, the technique used is the main finding which make the result of the study not convincing compared to worldwide agreements of MB2 It can be improved and focused on the type of technique used | Thank you for your valuable comments. The section was revised. | Page 12, lines 286-92. |
| **Reviewer #2:** | | |
| A good study has been done and most of the subjects that should be done in systematic review studies such as publication bias, risk of bias, heterogeneity, subgroup analysis, etc. have been done. But in order to increase the quality of the article, the following items are suggested to be reviewed and corrected: | We are grateful. | - |
| 1- PRISMA 2020 framework should be used in the study. | Done, Thank you. | Figure 1 |
| 2- Due to the fact that the resolution of the graphs is low, it is suggested to save the images of the graphs with the tif extension. | Done, Thank you. | - |
| 3- It is better to perform sensitivity analysis for studies with a small sample size, such as Naseri's 2015 study (sample of 35 people). In such a way that these studies are removed and recalculate the pool effect. | Thank you for your valuable comment, done. | Page 5, lines 104-6.  Figure 4. |
| 4- The tool used to check the risk of bias seems to be the tool for evaluating the quality of studies, rather than risk. It is better to mention this and add the score of each study in table 1 and 2 (bibliographic table). | Done, thank you for your valuable comment. | Page 4, line 93.  Table 1 and 2. |
| 5- The bibliographic information table is incomplete; it is better to mention the type of study and the quality score of the study. | Done, Thank you. | Table 1 and 2 |
| 6- Several graphs and parameters have been used for some topics, such as heterogeneity. It is better to report only the Galbraith graph for this topic. | Thank you for your comment, the figure 4 (Baujat plot) was deleted. | - |
